# Community-engaged Systems for Population Health Improvement: A Novel Approach to Improve Diabetes Outcomes in Rural Communities

**DOI:** 10.1007/s10900-024-01376-z

**Published:** 2024-07-09

**Authors:** Kristin Pullyblank, Marisa Rosen, Christopher Wichman, Ann E. Rogers, Melissa Baron, David A. Dzewaltowski

**Affiliations:** 1 Bassett Research Institute, Center for Rural Community Health, Bassett Medical Center, Cooperstown, NY USA; 2 College of Public Health, Department of Health Promotion, University of Nebraska Medical Center, Omaha, NE USA; 3 College of Public Health, Department of Biostatistics, University of Nebraska Medical Center, Omaha, NE USA

**Keywords:** Diabetes management, Health promotion, Complex systems, Rural, Process improvement, Community engagement

## Abstract

**Background:**

Approaches to prevent and manage diabetes at a community population level are hindered because current strategies are not aligned with the structure and function of a community system. We describe a community-driven process based on local data and rapid prototyping as an alternative approach to create diabetes prevention and care management solutions appropriate for each community. We report on the process and provide baseline data for a 3-year case study initiative to improve diabetes outcomes in two rural Nebraska communities.

**Methods:**

We developed an iterative design process based on the assumption that decentralized decision-making using local data feedback and monitoring will lead to the innovation of local sustainable solutions. Coalitions act as community innovation hubs and meet monthly to work through a facilitated design process. Six core diabetes measures will be tracked over the course of the project using the electronic health record from community clinics as a proxy for the entire community.

**Results:**

Baseline data indicate two-thirds of the population in both communities are at risk for prediabetes based on age and body mass index. However, only a fraction (35% and 12%) of those at risk have been screened. This information led both coalitions to focus on improving screening rates in their communities.

**Discussion:**

In order to move a complex system towards an optimal state (e.g., improved diabetes outcomes), stakeholders must have access to continuous feedback of accurate, pertinent information in order to make informed decisions. Conventional approaches of implementing evidence-based interventions do not facilitate this process.

Diabetes remains a public health priority, affecting 11.3% of the United States (US) adult population. As of 2020, the Centers for Disease Control and Prevention (CDC) estimated that the annual direct and indirect costs of diabetes was $327 billion [1]. Over the past four decades, individuals living within rural areas have emerged as a US National Institutes of Health defined population with health disparities because they experience comparatively worse diabetes outcomes than their urban counterparts [2–5]. The drivers of rural health disparities can be attributed to the complex interaction of many individual and systemic factors such as demographics, political power, geography, resource availability, and culture [6–8]. While there have been a myriad of attempts to improve both rural and urban diabetes outcomes, we continue to see an increasing prevalence of diabetes at the population level [9].

Population health is defined as the “health outcomes of a group of individuals, including the distribution of such outcomes within the group” (p. 381) [10]. Those interested in improving population health aim to understand the drivers of disparities, which could then be targeted by various strategies to address the historical and current unequal provision, production and distribution of social, political, economic, and environmental resources [11]. We argue that the historical and current approaches to population health problems have had muted success due to a misalignment between the structure of the social, political, economic and environmental system under consideration and the strategies implemented to address health improvement.

If we consider the management of population health of a group to be all patients of a health care system, i.e., the managed patient population, the delivery of health care service is typically described by a set of structural resource inputs, workflow processes, service outputs, and outcomes [12]. This model of the healthcare system is a closed system with one organization centrally controlling both the provision (the intervention) and the production (how it is implemented) of services in that system. The healthcare system is incentivized when it meets certain standards of care, quality and cost metrics [13]. Therefore, healthcare teams coordinate care within the system to manage and treat their individual patients with the goal of meeting these standards. One current strategy to meet this goal is to implement evidence-based clinical interventions. For example, the American Diabetes Association (ADA) guidelines recommend that continuous glucose monitoring should be offered to individuals with diabetes who require multiple daily injections as this strategy has been shown to improve diabetes-related outcomes [14]. Another current strategy is developing system processes to improve performance on quality metrics, such as the Diabetes INSIDE initiative, a quality improvement program involving community-integrated approaches and multidisciplinary care team coaching. This centralized, process-based approach has demonstrated improvements in A1c among a population of an entire healthcare system [15].

However, if we consider the management of population health of a group to be all people and organizations within a rural community geographic area, we are now working with a more complex and dynamic open system. In other words, the system spans across many places where people live, learn, work, play, and receive services with many different organizations with authority to manage those settings (e.g., healthcare systems, for-profit firms, not-for-profit community organizations, and local government agencies). For community social, economic and political systems, there is no central governing mechanism for non-infectious disease prevention and management. Instead, there are multiple decision centers (e.g., school, household, rec center, town council) each of which retains some independence. The decisions about what services to produce and how to produce them are made by separate entities within that system. Feedback from the outcome of these decisions determines the success or failure of that intervention as well as further decisions regarding it (e.g., a new walking trail that is frequently used by schoolchildren encourages the expansion of the trail and addition of amenities). In sum, the system characteristics of the population targeted (e.g., managed care population vs. entire community) influences the success of various approaches to improve population health because of the way in which they are coordinated.

Healthcare practitioners recognize the important contribution of various community settings in sustaining their patients’ health and have attempted to coordinate care with community social service organizations, public health, centers of worship, among others. The term community engagement (CE) has been used to describe this process of working collaboratively with groups of people who are connected by geography, special interests, or similar situations to address issues affecting their well-being [16, 17]. The practice of CE by a health care system typically involves forming a small group of stakeholders called an advisory committee or multi-sector coalition and following a CE process “of developing relationships that enable stakeholders to work together to address health-related issues and promote well-being to achieve positive health impact and outcomes” (p. 12) [18].

Although there are an array of CE models, there is a gap in the literature identifying the fit of the population health management strategy with the type of system to be managed (e.g., centralized or free-market). Current CE strategies to improve population health at the community level have largely been adopted from population health management strategies used within healthcare system patient management. This centrally controlled healthcare organization quality improvement approach of defined standard evidence-based practices (service provision), implemented through top-down performance improvement initiatives (service production) may use a community stakeholder group or multi-sector coalition. While this approach can be effective in a more controlled, within-organization system, these population health management design characteristics are not the optimal approach in a decentralized, complex community system that operates in a self-organized fashion, often based on market principles. First, community organizations are not accountable to the healthcare system and the incentives provided by the healthcare system are unlikely to alter community-based organizations’ approach to work. Second, conventional CE approaches involve an inherently long community needs assessment and planning period in an attempt to centrally coordinate the system. For example, The US Affordable Care Act requires not-for-profit hospitals to conduct a community health needs assessment every three years and to adopt an implementation strategy. By the time the intervention is finally implemented in such a complex, fluid system, it may be perceived as having little applicability to current conditions. Third, conventional health system CE approaches provide very little feedback (data) as to how the intervention is moving population health outcomes; feedback is instead limited to the reach of the intervention and any individual level changes. And, even if feedback is obtained it is based on needs and plans often generated two years prior. In brief, the current within-organization population health management CE design strategies may not appropriately align with managing whole community systems.

The coordination of *rural* communities for population health improvement involves additional challenges. First, rural communities are structurally different in input resources, existing processes for the provision, production, and distribution of services, and demanded services from the population compared to non-rural communities. Yet there are very few defined interventions or practices that were initially developed within rural communities [19]. Centrally controlled health care systems are often not located in rural areas, so the information received by decision-makers often lacks context and salience. In addition, the nature of being a rural community means that existing healthcare resources are generally more scarce, and therefore the conventional strategies described above may have less applicability in these communities [20, 21]. Finally, prevention and management of noninfectious disease in and of itself is much more dependent on resources within complex community systems compared to problems that can be addressed by the healthcare system sector alone [22].

Due to the dynamic nature of community systems and the decentralized self-organizing coordinating structure, it may not be possible to develop interventions that can be generalizable. However, we posit that it is possible to develop a generalizable set of design characteristics and a *standardized community action process* so communities can manage population-level solutions on their own. Within the community economic development literature, this general approach is labeled community-driven development where community members control resources and decision-making [23].

By taking a community-driven and data-driven process-based approach to impact the structure and function of the system in lieu of scaling an intervention-based approach within a system [24], we account for the dynamic nature of a complex community social, economic, and political system. The process facilitates local data to be fed back into the community, so that decisions can be made to move the system toward improved outcomes. In essence, we are flipping the implementation paradigm. Instead of tailoring evidence-based interventions for a particular community, we take a rapid cycling entrepreneurial high risk/high reward systems approach akin to what occurs in a free-market or self-organized system, to encourage the creation of strategies that will work under current system constraints. This approach has shown promise in several community-driven projects targeting childhood obesity prevention through physical activity and healthful eating [25–28]. The purpose of this paper is to describe the design and initial baseline findings for a local community-driven effort to improve diabetes outcomes among adults in two rural Nebraska communities.

## Methods

### Design

Diabetes On Track is a three-year (June 2022-June 2025) community case-study initiative funded by the Diabetes Care Foundation of Nebraska with the overarching goal of improving diabetes outcomes among rural Nebraskans. In addition to the community-driven effort described here, two parallel efforts are under way: the first involves transforming rural healthcare settings through a more traditional community engaged approach; and the second involves building a communication and referral pathway between community and healthcare systems. These efforts are described separately and employ strategies that align with the social system under consideration (e.g. managed care population). The project was considered quality improvement and exempt from continuing review by the University’s Institutional Review Board.

### Community Characteristics

In late 2021, two rural communities were identified and invited to participate as project sites based on the following inclusion criteria. The identified communities had to include a family practice that was connected to Nebraska Medicine’s electronic health record (EHR). One of the communities was to have a population between 20,000 and 25,000 with higher commuting patterns, while the other community was to have a population between 2,500 and 5,000 with lower commuting patterns. These parameters would allow the research team to investigate how the process worked in two different sized rural communities. Community participation was contingent upon a signed data use agreement between the university and the healthcare system.

Community A has a population of 24,691, with 94.1% identifying as white, and 87.7% identifying as non-Hispanic (United States Census Bureau, 2020). The community is associated with two different Rural-Urban Commuting Area (RUCA) codes: 4 (micropolitan area core) and 5 (micropolitan high commuting). The county’s Rural Urban Continuum Code (RUCC) is classified as 4 (nonmetro; urban population of 20,000 or more, adjacent to a metro area) [29, 30]. The community has five family/internal medicine clinics, and one hospital with a comprehensive diabetes program. The median household income is $57,783, and 11.7% of the county population is considered to be living in poverty. The regional (i.e., multi-county) public health department responsible for Community A has a physical office located in Community A.

Community B has a population of 5,840, with 93% identifying as white, 91.4% as non-Hispanic. The community has two RUCA classifications: 7 (small town core) and 10 (rural area). The county has a RUCC of 6 (urban population of 2,500–19,999, adjacent to a rural area) [29, 30]. The median household income is $40,339 with 19% living in poverty. Similar to Community A, the regional public health department responsible for Community B, has a physical office location within Community B (United States Census Bureau, 2020).

The target group includes those adults living in these communities who are at risk for developing prediabetes or diabetes based on age (age ≥ 19) and BMI criteria (age 30–70, BMI ≥ 25), or who already have a diagnosis of diabetes.

### Coordination System Design Characteristics

As described above, systems can be coordinated to produce and provide resources in different ways. While health improvement strategies such as the implementation of evidence-based practices and coordinated planning can be effective in centrally controlled systems, improvement strategies using an iterative, entrepreneurial process align with the decentralized nature of community systems and provide the opportunity for coordination across sectors rather than top-down implementation of one strategy. The three key elements of our Diabetes on Track community-driven population health coordination model are the components that are necessary in any successful self-organized community system [23]. These include a local data and monitoring feedback system, a community innovation hub, and a community action process. This approach will foster a rural community entrepreneurial “trial and error” learning system for improved community-level diabetes related health outcomes.

#### Local Data Monitoring and Feedback System

Six core population health measures extracted from aggregate EHR data in each community are used as the primary outcome measures (Table 1). These measures are routinely provided to the coalitions within each community to drive the decision-making process, as described in more detail below. Diabetes-related metrics include the prevalence of adults (30–70) at risk for prediabetes and diabetes (BMI ≥ 25); the percentage of those adults (30–70) at risk (BMI ≥ 25) who have been screened for prediabetes or diabetes in the past 36 months; the percentage of adults (30–70, BMI ≥ 25) who have a diagnosis of prediabetes among those who are screened; the percentage of adults (≥ 19) who have confirmed diabetes; the percentage of adults (≥ 19) with confirmed diabetes who have a documented A1c in the medical record in the last 12 months; and the percentage of adults (≥ 19) with confirmed diabetes and a yearly A1c documented, whose last documented A1c was > 9. 

**Table 1:** Population Health Outcome Measures

Indicator	Measures
Prediabetes Metrics	Active* patient at risk for prediabetes (age 30–70, BMI ≥ 25)
	Among those at risk, who has been screened for prediabetes or diabetes (A1c in EHR within last 36 months)
	Among those screened, with confirmed prediabetes (A1c 5.7–6.4%)
Diabetes Metrics	Adults age 19 and older with diagnosis of diabetes (type 1 or type 2) in the EHR based on the EHR problem list
	Among those 19 or older with a diagnosis, A1c in EHR within last 12 months
	Among those 19 or older with a diagnosis and A1c in EHR, within last 12 months with last HbA1c > 9

* Active patient is defined as a patient who has been seen in the clinic within the last three years

The presence of local data presents the opportunity to decrease uncertainty in a system and motivate individuals to use the data to make decisions that improves the situation for themselves and for their communities [31]. Local data are also important for timely feedback in the system as data allow local community members to see if and how an intervention is moving the system toward a different outcome (e.g. improved diabetes screening rates). Most conventional interventions rely on state or regional data from public health surveillance systems. Unfortunately, for many rural communities, much of this data is suppressed due to privacy concerns, which may mask important community-level indicators or lead to overgeneralizing health concerns for any particular community [19, 32]. Our study uses EHR data from the health system within the community. Even though not all primary care practices were involved in the community coalition, aggregate medical record data are available for one-third of the community population in both communities. This subset of data is both relatable and meaningful for the community members as they examine trends in prediabetes prevalence, diabetes prevalence and those who had been screened in the last year for prediabetes or diabetes. In addition, as the project progresses, the community coalitions will be defining and collecting implementation metrics and local community-defined measures of success around reach and impact of the different diabetes care, education, prevention and screening services they are designing.

#### Community Innovation Hub

The purpose of the community innovation hub (“hub”) is to act as a local learning network community where stakeholders from diverse sectors exchange knowledge and ideas and work together to solve common issues in order to improve population health outcomes. In this case, the hubs in each community were asked to address ways to improve diabetes services for people living with diabetes or at risk for developing diabetes. Unlike coalitions that are developed in more conventional CE models (e.g., Collective Impact) [33] our hub is not charged with delivering a needs assessment, implementation plan, or implementing any specific intervention [33]. Rather it acts as the coordinating hub of local community stakeholders (individuals and organizations) who are invested in the health and wellness of the local population. Hub members will be designing multiple prototypes and working with their home agencies or other community members to implement them, thus moving the entire system towards health improvement.

#### Community Action Process

Through the use of a rapid cycle, quality improvement process of Investigate-Design-Practice-Reflect (IDPR), the coalition members develop prototypes (programs, policies, or practices) to implement in their community. The IDPR process parallels the fundamental functions required for a feedback control system: investigate (sensor), design (controller), practice (effector), and reflect (system feedback) [34]. Structurally, IDPR is similar to the iterative Plan-Do-Study-Act (PDSA) cycle but it relies on investigating the local conditions or system state as its initial step, so the community has a better idea of the boundaries, assets and constraints of the system. In addition, because we are working under the assumption that there is no certainty in inputs or outcomes due to the complex dynamic nature of a community system, the IDPR process asks participants to initially develop a simplified version of the product to get some feedback on how it should work (e.g., organizing one cooking class before developing an entire year-long series). Using that feedback, participants continue to refine and redesign their prototypes rapidly. Based on a hub-developed data-driven headline that guides their work, hub members have the freedom to design and implement multiple prototypes to ascertain how each one works under the constraints of the system. This trial-and-error learning approach is analogous to the process that occurs in a free market where the feedback to produce a service or good is immediately provided by consumers of that service or good.

### Local Capacity Building Process and Timeline

The capacity-building process for local community-driven population health efforts include three phases that, depending on local development, roughly correspond to three years: Phase 1- baseline infrastructure development; Phase 2 – community action process capacity development; Phase 3 – Transition to sustainable community-driven population health management.

#### Phase 1: Baseline Infrastructure Development

The technical support and training (TST) team identifies a coordinating unit (CU) of the innovation hub (in this study, the local health department (LHD) in both communities was identified for this role). The CU is responsible for convening community partners, facilitating information sharing, and coordinating the implementation of any strategies. Meanwhile, the TST team (in this case, our research team from UNMC) develops the capacity to deliver a local data monitoring and feedback system. For this project, this included establishing a data sharing agreement with Nebraska Medicine so aggregate EHR data from the community clinics could be extracted throughout the project period, as well as developing a protocol for the dissemination of stakeholder and community level surveys. In addition to managing the data and feedback monitoring system, the TST team assists with the local coordination of the project.

#### Phase 2: Community Action Process Capacity Development (Current Phase of Project)

In-person hub meetings occur periodically (in our study, they occur monthly) with a web-based platform available for those unable to attend in person. The TST team guides the participants through the IDPR process using a facilitator’s guide developed for this purpose. The innovation hub moves through the process at its own pace. The IDPR process has been discussed in more detail elsewhere [35]. Elements included in the process are described in Table 2.

**Table 2:** Investigate-Design-Practice-Reflect process elements and associated activities

Element	Guiding Question	Resources/Tools
Investigate	What is our community diabetes care management, prevention, and education landscape?	• Place-based mapping process • Diabetes navigation pathway map • Asset mapping process • Social and power network mapping process • Social network map • Community data reports • Data summary sheet or community dashboard
Design	How do we build a coordinated and sustainable system for local residents to receive efficient screening, referral, and engagement of pre-diabetes and diabetes across behavior settings with our communities?	• All Investigate activities • Community headline • Community roadmap • Prototype in progress
Practice	What did we implement along the Diabetes Care Pathway to impact individuals in our community?	• Data summary sheet or community dashboard • Partner reflection • Community prototype
Reflect	How did we impact the Diabetes Care Pathway?	• Data summary sheet or community dashboard

The initial community data report collected through the data monitoring and feedback system is brought to the first coalition meeting to guide the initial investigative phase of the process. In our study, we used the following Behavioral Risk Factor Surveillance System (BRFSS) county-level metrics to establish a baseline for various health behaviors: percentage of adults aged 18 years and over reporting no leisure time physical activity and percentage of adults aged 18 years and older that reported a BMI ≥ 30. Surveillance data is also used to collect information on the wellness landscape. In our study, the following metrics were collected from the community needs assessment as well as the county-level BRFSS: access to nutrition, physical activity, and weight management and education programs within an hour from the home; barriers to self-care and care for immediate family; percentage of adults who could not see the doctor in the past 12 months due to cost, and percentage of adults 18–64 years old with no health care coverage. The presence of this data helps facilitate the IDPR process including assisting the community in determining what aspect of the diabetes care, education, screening and prevention pathway they wanted to focus on addressing first.

#### Phase 3: – Transition to Sustainable Community-Driven Population Health Coordination

Several conditions must be in place for the TST team to step back and transition system coordination to the community hub. First, the data monitoring system must be robust enough that there is bidirectional information exchange, allowing for community members to make decisions about where to focus efforts, what additional prototypes may need to be developed or adapted, and articulate measures of success. Second, people involved in the hub must feel comfortable working with one another, and comfortable in supporting the involvement of other community members. Hub members need to recognize their own autonomy to move forward in a way that is most appropriate for the community. Finally, the hub must feel comfortable with the IDPR process. This means that the hub demonstrates its capacity to develop and trial prototypes quickly (e.g., without an extended planning period), recognizing that it is the implementation of these prototypes that will give valuable feedback to the implementers, allowing them to adapt the prototype as necessary. Self-report survey scores on trust and autonomy (described below) will provide insight into a community hub’s readiness to transition.

### Evaluation Plan

There are three components of the evaluation plan: examine the change in core population health outcome metrics; note how the structure and function of the community hubs changed over time; assess output from the communities’ rapid prototyping processes. Each component will be described below.

The six core outcome measures (Table 1) will be extracted on a biannual basis via the EHR. These data will be shared with the research team in a deidentified, aggregate manner. Significant change over time will be calculated using repeated measures analysis of variance. Baseline values are described in Table 3.

**Table 3:** Baseline Population Outcome Results (6/15/2022):

Metric	Numerator	Denominator	Community A No./Total No. (%)	Community B No./Total No. (%)
At risk for prediabetes	All patients age 30–70, BMI ≥ 25	All patients age 30–70	3599/5476 (65.7)	2133/3220 (66.2)
At risk, who were screened for prediabetes	All patients age 30–70, BMI ≥ 25, HbA1c within last 36 months	All patients age 30–70, BMI ≥ 25	1263/3599 (35.1)	261/2133 (12.2)
Screened, with confirmed prediabetes	All patients age 30–70, BMI ≥ 25, HbA1c within last 36 months, most recent HbA1c between 5.7% and 6.4%	All patients age 30–70, BMI ≥ 25, HbA1c within last 36 months	502/1263 (39.7)	72/261 (27.6)
Diagnosed DM among all adults	Adults age 19 and older with diagnosis of diabetes (type 1 or type 2) in the medical record	All patients age 19 and older	1339/10,575 (12.7)	451/6617 (6.8)
Among diagnosed with DM, HbA1c in chart within last 12 months	Adults age 19 and older with diagnosis of diabetes (type 1 or type 2) in the medical record with HbA1c within last 12 months	Adults age 19 and older with diagnosis of diabetes (type 1 or type 2) in the medical record	1127/1339 (84.2)	359/451 (79.6)
Among those with A1c within last 12 months, most recent HbA1c > 9	Adults age 19 and older with diagnosis of diabetes (type 1 or type 2) in the medical record with HbA1c within last 12 months, with last HbA1c > 9	Adults age 19 and older with diagnosis of diabetes (type 1 or type 2) in the medical record with HbA1c within last 12 months	162/1127 (14.4)	47/359 (13.1)

Process metrics related to the structure of the hub include stakeholder composition, and members’ connection with the community as defined by whether stakeholders live in the community and for how long they have resided in the community. Indicators of the hub’s function include members’ average level of satisfaction with the diabetes care and education landscape in the community (measured on a 1–5 Likert scale with 1 = “not satisfied” and 5 = “extremely satisfied”), and average level agreement about readiness for collective action (measured on a 1–5 Likert scale with 1 = “strongly disagree” and 5 = “strongly agree”). In addition, stakeholders are asked questions to assess level of individual and collective trust and autonomy (measured on a 1–5 Likert scale with 1 = “strongly disagree” and 5 = “strongly agree”). Prior research has indicated that trust is essential for enabling cooperative and adaptive behavior within organizations and networks, reducing conflict, and decreasing transaction costs [36]. We are also measuring autonomy because this is an essential element of motivating an individual to reach long-term goals (e.g. improving the wellness landscape) [37], and lack of autonomy has been cited as a barrier to coalition success [38]. These measures will be collected annually. Baseline values are found in Table 4.

**Table 4:** Community Hub Characteristics

Indicator	Community A	Community B
**Satisfaction with diabetes landscape**		
How satisfied are you with the variety of places adults can go for diabetes screening services in the community?	2.8	1.6
How satisfied are you with the variety of places adults can go for diabetes prevention services (e.g., nutritional, physical activity, education, etc.) in the community?	2.6	1.9
How satisfied are you with the variety of places adults can go for diabetes care management services in the community?	2.9	1.7
**Agreement with readiness for collective action**		
The community culture demonstrates a collective commitment to support community wellness initiatives.	3.6	3.3
The local political and social climate seems to support starting a collaborative community initiative like this one.	3.6	3.7
Organizations in our community have a history of working together on wellness initiatives.	3.8	3.8
**Individual trust**		
I trust others in the community coalition to work towards community diabetes objectives	4.0	3.9
I trust the diabetes on track team to work towards community diabetes objectives	4.2	4.1
I respect other people involved in this community coalition	4.2	4.3
**Individual autonomy**		
I have choices and options about my role and how I contribute to the community coalition	3.9	4.0
**Collective trust**		
Community coalition members trust each other to work towards community diabetes objectives	3.7	3.9
Community coalition members trust the Diabetes on Track team to work towards community diabetes objectives	3.9	4.0
**Collective autonomy**		
The community coalition has choice and options about how to impact the diabetes prevention and care landscape of the community.	3.7	3.7

The unique nature of our community population health improvement strategy based on data-driven decision-making means that we cannot determine some of the evaluation metrics a priori regarding the community rapid prototyping process. Instead, each prototype (e.g., event, change in process) will have its own evaluation metrics as determined by the coalition members. The research team will help support analysis of data. It is likely that the data collected will be analyzed descriptively. Qualitative data will most likely be evaluated using content analysis [39] or qualitative description [40]. We will use descriptive statistics to describe quantitative data and bivariate inferential statistics to measure any differences between groups (e.g., pre/post). Importantly, the data will need to be regularly presented back to the stakeholders in a manner that is useful and relevant. In addition, we will be tracking the number of policy, systems and environmental (PSE) changes that occur relevant to the diabetes landscape during the project period. These results will be reported after the end of the project period.

## Results

### Baseline Core Outcome Measures

At baseline, prediabetes risk in both communities was around two-thirds of the adult population between the ages of 30–70, as determined by having a BMI of at least 25 kg/m^2^. (Table 3). Of those who were at risk and had been screened for prediabetes, around a third of people had confirmed diabetes.

In addition, the prevalence of diabetes within Community A was nearly 13%, while it was around 7% in Community B. About 9.8% of the adult population of Nebraska has a diagnosis of diabetes [41]. Of those adults with confirmed diabetes in the two communities, approximately 80% had an A1c documented in the medical record in the last 12 months. Of those who had a documented A1c within the last 12 months, 14.4% had the most recent A1c > 9 in community A, and 13.1% had values > 9 in community B.

### Baseline Community Innovation Hub Characteristics

At the initial hub meetings, community A had 18 stakeholders representing 11 organizations (plus two community members not affiliated with an organization) while community B had 5 stakeholders representing 3 organizations. About three-quarters (73.7%) of community A’s hub participants lived in community A (average years in town was 21.6). Meanwhile, 28.6% of community B’s hub participants lived in community B (average years in town was 10).

Baseline satisfaction with the diabetes care, management and education landscape was higher in Community A than in community B. However, readiness for collective action was comparable between the two communities. Both communities felt they had a strong history of collaborating with community partners on wellness initiatives. Individual and collective trust levels were similar between communities. Community A had higher levels of agreement with the individual trust statements versus the collective trust statements, whereas this was not noted in Community B. Both community A and Community B agreed more strongly with the individual autonomy statement than with the collective autonomy statement.

## Discussion

Numerous efforts have attempted to tackle the diabetes epidemic in rural US communities using population-based approaches [6, 42, 43]. Most of these efforts have had limited impact on population health outcomes at the community levels for a couple of reasons. First, the population under consideration is really a managed care population, and not the community population at large [42, 44]. Second, the interventions or suite of programs implemented are implemented in a linear, reductionist manner that is inconsistent with how a complex system is coordinated, leading to a lack of scaling and sustainability and lack of impact on community-level outcomes [45].

Kickbusch and Gleicher clarify that when faced with “wicked” public health problems, it is important that public health interventions work towards creating a resilient, adaptable system [46]. This perspective supports the need for a paradigm shift that the Diabetes On Track initiative is responding to with our local, data-driven, rapid prototyping process. First, our initiative understands that in order to change population-level health outcomes, the focus should be on changing the social, economic, and political system structure rather than only changing an individual’s behavior. Second, in order to develop solutions in a complex system, we must engage in strategies that are akin to how the system functions. For free-market community systems, it is important to develop and trial multiple prototypes simultaneously and receive feedback as to whether the prototype affected the local system in a positive fashion. Such a model copes with unique local system characteristic and disruptions and creates redundancy in and therefore resiliency in a system where disruption (e.g. change in leadership, change in policies, global pandemic) is the norm [47]. As such, a local data-driven, rapid prototyping process is essential to aligning with local system functions.

Finally, incorporating a continuous local data monitoring and feedback system into the process gives community members valuable information about how the system is responding to any prototype. Hub members are able to interpret the data coming back from their interventions and decide how to adjust their strategies.

In the Diabetes On Track project, community-level baseline data indicate that in both communities, two thirds of the adult population is at risk for prediabetes but only a small portion (35% in community A and 12% in community B) have been screened for prediabetes. In addition, community members, particularly those in community B, are not satisfied with the number of community resources available for diabetes prevention, education and management. These data have led both communities to make a decision to focus on shifting the system to increase awareness of screening rates. Instead of focusing on a predetermined set of interventions that may be limited in reach and appeal to a community at a particular time and place, Diabetes On Track participants will create a diversity of community-informed solutions through an iterative data-driven process.

This community case study is not intended to test whether this process is more effective at improving population-level diabetes outcomes than conventional approaches. Rather, the intent is to determine if implementing the process is feasible. Future community-randomized controlled trials will need to assess if shifting the paradigm to a process-based approach facilitates improvement in population-level diabetes outcomes.

## Conclusion

The Diabetes On Track initiative is an innovative community effort designed to create a sustainable process through which a community acts as an agent for change in diabetes outcomes. Based within a theoretical framework that is fundamentally different from public health’s standard approaches of implementing evidence-based practices, Diabetes On Track leverages the local knowledge, data, and assets of a community to rapidly develop potential solutions that are resilient in an ever-changing community landscape.

## Data Availability

The data that support the findings of this study are not openly available due to the ongoing nature of the study but are available from the corresponding author upon reasonable request. Data are located in controlled access data storage at University of Nebraska Medical Center.
